# The underlying mechanism of mirror visual feedback in stroke. In reply to: challenging the electrophysiological interpretation of mirror visual feedback in stroke

**DOI:** 10.1016/j.cnp.2025.08.007

**Published:** 2025-09-09

**Authors:** Jinyang Zhuang

**Affiliations:** Department of Rehabilitation Medicine, Ruijin Hospital, Shanghai Jiao Tong University School of Medicine, Shanghai 200025, China

In the domain of upper extremity (UE) rehabilitation following a stroke, mirror visual feedback (MVF) is widely used due to its simplicity, flexibility, and cost-effectiveness. The mechanism of MVF is associated with mediating the maladaptive neurophysiological process, thus restoring the sensorimotor function of UE poststroke. Our study showed that MVF reduced activity in contralesional motor and parietal cortices and enhanced hemispheric balance in the low mu band during early stroke recovery (Zhuang et al., 2025a). This finding invited critical scrutiny and raised questions regarding its consistency with existing researches, suggesting the possibility of bias (Sathian et al., 2025). The challenging idea posited that MVF should enhanced the cortex activities, while suggesting that the low mu band was not the biomarker of MVF. In addition, the scholars indicated that the MVF might not facilitate the hemispheric balance. Nevertheless, the precise neural mechanisms underlying MVF remain inadequately understood. For instance, the perspective that MVF activated mirror neurons had been contested by a prior study, which suggested that MVF supported post-stroke motor recovery by engaging brain regions associated with attention and cognition, rather than mirror neurons (Michielsen et al., 2011).

In clinical practice with MVF, a mirror is placed in the midsagittal plane to reflect the unaffected limb onto the affected one, involving motor observation, imagery, and execution. Visual observation is crucial for the efficacy of MVF. A prior study employing dynamic causal modeling analysis showed that MVF activated the motor cortex through the contralesional action observation network, with the intact parietal cortex playing a key role (Saleh et al., 2017). This finding supported a causal relationship between the therapeutic effects of MVF and the mechanism of visual observation. Patients with stroke often neglect their paralyzed limbs, a phenomenon referred to as “learned non-use.” MVF directs attention to the affected limbs and may reactivate dormant motor areas by closing the visual feedback loop, thereby enhancing the motor function of UE (Ramachandran and Altschuler, 2009). Recently, our study showed that mirror visual observation, when isolated from MVF, could prime motor recovery of UE poststroke, thereby underscoring the significance of visual observation in the context of MVF (Zhuang et al., 2025b).

The Event-Related Desynchronization (ERD) of electroencephalogram (EEG) has emerged as a prominent indicator for investigating the mechanisms underlying MVF. MVF may affect ERD of different frequency bands and may involve one or both brain hemispheres (Bartur et al., 2018, Lee et al., 2015). MVF could activate sensorimotor cortex within the mu band of ERD (Lee et al., 2015). The mu band is categorized into low mu (8–10 Hz) and high mu (10–12 Hz) bands. Our recent findings showed that MVF affected motor and parietal cortices within low mu band which mainly associated with motor observation (Frenkel-Toledo et al., 2014), suggesting that the mechanism of MVF was related to mediating visual observation (Zhuang et al., 2025a). MVF was also showed to reduce ERD in both hemispheres within the low-beta EEG oscillations, a more pronounced reduction on the unaffected side, thereby balancing hemispheric activity (Bartur et al., 2018). One preview study using the lateralization index (LI) also demonstrated that MVF promoted the balance of bilateral hemispheres activities (Lee et al., 2015). Based on above studies, MVF may support stroke rehabilitation by suppressing contralesional hemisphere activation and promoting the balance of hemispheric activities.

Currently, there is no consensus on whether low beta ERD is more sensitive to MVF induced cortical activation than the mu band. Variability among individual patients, including differences in lesion laterality, lesion types, and stroke phases, may introduce biases in studies investigating the balance of bilateral hemisphere activity. To address these limitations, a large-sample cohort study utilizing EEG should be conducted. Additionally, future researches can employ functional Magnetic Resonance Imaging (fMRI) and Transcranial Magnetic Stimulation (TMS) to validate EEG results. We recommend collaborative efforts among research teams to explore the precise mechanisms underlying MVF and to advance the clinical application and development of this technology in stroke rehabilitation.
